# The utility of superficial abdominal reflex in the initial diagnosis of scoliosis: a retrospective review of clinical characteristics of scoliosis with syringomyelia

**DOI:** 10.1186/1748-7161-5-17

**Published:** 2010-08-26

**Authors:** Takahito Fujimori, Motoki Iwasaki, Yukitaka Nagamoto, Hironobu Sakaura, Kazuya Oshima, Hideki Yoshikawa

**Affiliations:** 1 Department of Orthopaedic Surgery, Osaka University Graduate School of Medicine, 2-2 Yamadaoka, Suita, Osaka 565-0871, Japan

## Abstract

**Background:**

With increasing use of magnetic resonance imaging (MRI), underlying syringomyelia is increasingly found in patients with presumed idiopathic scoliosis. To determine the indications for MRI in the differential diagnosis of scoliosis, several clinical characteristics of syringomyelia have been reported. Neurological signs, particularly abnormal superficial abdominal reflex (SAR), are important in establishing the initial diagnosis of scoliosis. However, the prevalence of abnormal SAR in patients with scoliosis and the sensitivity of this sign in predicting syringomyelia are not well known. We aimed to determine the diagnostic utility of SAR and other characteristics of syringomyelia in patients with scoliosis.

**Methods:**

We reviewed the medical records of 93 patients with scoliosis, 90 of whom underwent corrective surgery. All patients underwent MRI to determine the presence of syringomyelia. Mean age at surgery was 12.5 years. Abnormal SAR was defined as unilateral or bilateral absence or hyporeflexia of SAR. We calculated indices of diagnostic utility of abnormal SAR for non-idiopathic scoliosis and for syringomyelia. Abnormal SAR, left thoracic curve pattern, gender, and curve flexibility were compared between scoliosis with syringomyelia and idiopathic scoliosis. Logistic regression analysis was performed with the existence of syringomyelia as the dependent variable and curve flexibility as the independent variable.

**Results:**

Abnormal SAR was observed in 20 patients (prevalence 22%). All 6 patients with myopathic scoliosis displayed bilateral absence of SAR. The sensitivity of abnormal SAR for non-idiopathic scoliosis was 38%, with 96% specificity, 90% PPV (positive predictive value), and 60% NPV (negative predictive value). Syringomyelia was identified in 9 of the 93 patients (9.7%); 8 of these had abnormal SAR. The sensitivity of abnormal SAR for syringomyelia in presumed idiopathic scoliosis was 89%, with 95% specificity, 80% PPV, and 98% NPV. Gender, abnormal neurological findings, and curve flexibility differed significantly between patients with syringomyelia and those with idiopathic scoliosis (P < 0.05). In the logistic regression model, the area under the receiver operating characteristic (ROC) curve was 0.79 and the cut-off value of curve flexibility for syringomyelia was 50% (P = 0.08).

**Conclusion:**

Abnormal SAR was a useful indicator not only for syringomyelia, but also for myogenic scoliosis.

## Background

Correct initial diagnosis of patients with scoliosis is important for decisions on treatment options. Misdiagnosis of scoliosis with underlying syringomyelia may result in complications following operative therapy or even brace therapy [1-3]. However, recent studies have reported that even patients with both scoliosis and syringomyelia have a small risk of neurological complications if they are neurologically normal or have a small syrinx [4-7]. Therefore, whether all patients with scoliosis should be examined by magnetic resonance imaging (MRI) remains controversial from a cost-effectiveness perspective [4,6-9]. The prevalence of underlying syringomyelia in cases of "idiopathic" scoliosis also differs between previous reports (range, 2-28%) [4,10-12]. Several indicators of syringomyelia have been reported in scoliosis patients: left thoracic curve [3,13-16], juvenile-onset scoliosis [11,15,17], rapid curve progression [3,18], pain [14], thoracic hyperkyphosis [19-21], male sex [5], and abnormal neurologic signs [4,5,9,13,22]. Of these indicators, neurologic signs, particularly an abnormal superficial abdominal reflex (SAR), is thought to be one of the most important clinical signs of syringomyelia[4,9]. In a study of 72 patients with scoliosis and atypical features, Morcuende reported that all patients with abnormal SAR had Arnold-Chiari malformations [9]. In a study of 327 patients with adolescent idiopathic scoliosis, Do emphasized the importance of abnormal SAR, even though the sensitivity and specificity of this sign was questionable [4]. Thus, although abnormal SAR has been regarded as an important neurological sign in the initial diagnosis of scoliosis, the prevalence of abnormal SAR and the diagnostic utility of abnormal SAR in predicting scoliosis with syringomyelia or other types of scoliosis has not been well known. We therefore examined the prevalence and diagnostic utility of abnormal SAR in all patients with scoliosis treated by a single spinal surgeon. In corrective surgery for scoliosis with syringomyelia, there is a potential risk of paraplegia: Overcorrection, tenuous blood supply, and changes in cerebrospinal fluid pressures are thought to be the causes of neurological complications [1,3,23]. In our experience, the curves of scoliosis with syringomyelia are likely to be more flexible than those of idiopathic scoliosis. However, few studies have reported differences in scoliosis curve flexibility relating to the presence or absence of syringomyelia. The purpose of this study was to determine the utility of abnormal SAR and curve flexibility in the initial diagnosis of scoliosis, particularly for scoliosis with syringomyelia.

## Methods

### Subjects

In this retrospective study we reviewed the medical records of 93 patients with scoliosis. Of these, 90 consecutive patients underwent corrective surgery for scoliosis and the other 3, who had syringomyelia, were followed non-operatively. Patients with adult scoliosis and degenerative scoliosis were excluded. Age was 0-3 years in 1 patient, 4-9 years in 15 patients, and 10-19 years in 77 patients. Mean age at surgery was 12.5 years (range, 3-28 years). Final diagnosis was idiopathic scoliosis in 46 patients, scoliosis with syringomyelia in 9, other neuropathic scoliosis in 3, congenital scoliosis in 9, myopathic scoliosis in 7, thoracogenic scoliosis in 5, neurofibromatosis in 3, Marfan syndrome in 3, and other symptomatic scoliosis in 8. "Idiopathic" scoliosis which means idiopathic-like scoliosis was defined as idiopathic scoliosis and scoliosis with syringomyelia. In the present study 55 patients were regarded as "idiopathic" scoliosis.

Of the 9 patients with syringomyelia, 6 underwent corrective surgery with uneventful recoveries and the other 3 patients with mild deformity were treated with corrective braces. Five of the 6 patients undergoing corrective surgery underwent neurosurgery for syringomyelia and 2 of the 3 patients with brace therapy underwent neurosurgery (foramen magnum decompression: FMD or syringo-subarachnoid shunt: S-S shunt). Demographic data of the 9 patients with syringomyelia are summarized in Table 1.

**Table 1:** Demographic data of 9 patients with syringomyelia

Case	1	2	3	4	5	6	7	8	9
Age (years)	28	8	12	9	14	16	6	11	12
Gender	M	M	F	F	F	M	F	M	F
Abnormality	Chiari	Syrinx	Chiari	Syrinx	Chiari	Chiari	Chiari	Chiari	Chiari
Cobb angle (°)	90	60	105	85	71	56	(38)	(23)	(30)
Flexibility (%)	52	78	55	50	56	58	None	None	None
SAR	Bil hypo	R abs	L abs	Bil hypo	L abs	L hypo	L abs	L hypo	Normal
Convex side	rt T	rt T	rt T	rt T	lt T	rt T+ lt L	lt T-L	lt L	rt T
Corrective surgery	ASF	ASF	ASF + PSF	ASF + PSF	PSF	PSF	None	None	None
Neurosurgery	None	SS	FMD	SS	FMD	FMD+ SS	FMD	FMD	None

ASF, anterior spinal fusion; Chiari, Arnold-Chiari malformation with syrinx; FMD, foramen magnum decompression; lt, left; L, lumbar; PSF, posterior spinal fusion; rt, right; SAR, superficial abdominal reflex; SS, syringo-subarachnoid shunt; T, thoracic; T-L, thoracolumbar

### Neurological assessment

Before ordering MRI, a senior orthopedic spinal surgeon (M.I.) performed detailed neurological examinations, consisting of an evaluation of sensory and motor functions, deep tendon reflexes of the lower extremities and Babinski reflex, as well as SAR. To examine SAR, patients were positioned supine on an examination table with arms relaxed along the sides of the body and the skin was lightly scratched with a blunt pin in the plane of a dermatome from the outer abdomen toward the midline. Presence of SAR was defined as clear deviation of the umbilicus toward the test side. Absence of SAR was defined as no movement of the umbilicus. Hyporeflexia of SAR was defined as less pronounced deviation than in normal cases. Normal SAR was defined as clear deviation of SAR on both sides of the abdomen, and abnormal SAR was defined as absence or hyporeflexia of SAR on one or both sides of the abdomen (including unilateral hyporeflexia or absence and bilateral hyporeflexia or absence). In addition, we calculated sensitivity, specificity, positive predictive value (PPV), and negative predictive value (NPV) of SAR for non-idiopathic scoliosis and for scoliosis with syringomyelia.

### Radiograph assessment

Posteroanterior (PA) and lateral radiographs of the entire spine were taken in a standing position to evaluate deformities. Curve flexibility was calculated using the following formula: (standing angle - traction or side bending angle)/standing angle × 100 (%). Flexibility was compared between operatively-managed patients with syringomyelia and those with idiopathic scoliosis. Logistic regression and receiver operating characteristic (ROC) analysis was performed with the existence of syringomyelia as the dependent variable and curve flexibility as the independent variable.

### Magnetic resonance imaging

A Signa Excite 1, 5_T system (General Electric Medical Systems, Milwaukee, WI, USA) was used to produce T1 and T2 weighted sagittal screening images of the cervical, thoracic and lumbar spine for all patients in the study, along with axial images of the craniocervical junction, cervicothoracic junction and thoracolumbar junction. All images were evaluated by a neurological radiologist, and by a spinal surgeon to identify hind brain and spinal cord abnormalities, recording the position of the conus medullaris and the presence or absence of syringomyelia and Arnold-Chiari malformations.

### Statistical analysis

Wilcoxon rank-sum test and Fisher's exact test were applied for statistical analysis using JMP 8.0 software (SAS Institute, Cary, NC, USA), as appropriate. All tests were evaluated as two-tailed. Values of P < 0.05 were considered significant.

## Results

### Abnormal SAR among all patients with scoliosis

Abnormal SAR were observed in 20 patients (prevalence 22%) (Table 2).

**Table 2:** Abnormality of superficial abdominal reflex (20 of 93 cases; 22%)

Abnormal SAR	Number of cases	Diagnosis
A: Bilateral absence	6	6 myopathic
		
B: Unilateral absence	6	4 syringomyelia
		1 thoracogenic (after abdominal surgery)
		1 myelomeningocele (after abdominal surgery)
		
C: Bilateral hyporeflexia	5	2 syringomyelia
		1 Duchenne muscular dystrophy
		2 idiopathic
		
D: Unilateral hyporeflexia	3	2 syringomyelia
		1 thoracogenic (after abdominal surgery)

SAR, superficial abdominal reflex

A: Bilateral absence of SAR.

All six patients with bilateral absence of SAR had myopathic scoliosis.

B: Unilateral absence of SAR.

Six patients had unilateral absence of SAR; 2 had absence of SAR on the side of abdominal surgery, and the other 4 patients had syringomyelia.

C: Bilateral hyporeflexia of SAR.

Five patients had bilateral hyporeflexia of SAR; one had muscular dystrophy, 2 patients had idiopathic scoliosis, and the other 2 patients had syringomyelia.

D: Unilateral hyporeflexia of SAR.

Three patients had unilateral hyporeflexia of SAR; one patient with thoracogenic scoliosis had hyporeflexia on the side of abdominal surgery, and the other 2 patients had syringomyelia.

Consequently, all 7 patients with myopathic scoliosis, 8 of 9 patients with syringomyelia and other 5 patients had abnormal SAR. The sensitivity of abnormal SAR for non-idiopathic scoliosis was thus 38%, with 96% specificity, 90% PPV, and 60% NPV (Table 3). There was a significant difference in the prevalence of abnormal SAR between patients with non-idiopathic scoliosis (38%) and those with idiopathic scoliosis (4%) (P < 0.0001).

**Table 3:** Abnormal SAR as a predictor of non-idiopathic scoliosis in all patients

	Non-idiopathic	Idiopathic	Total	
Abnormal SAR	18	2	20	PPV 90%
Normal SAR	29	44	73	NPV 60%
Total	47	46	93	
	Sensitivity 38%	Specificity 96%		

NPV, negative predictive value; PPV, positive predictive value; SAR, superficial abdominal reflex

### Abnormal SAR in patients with syringomyelia

**Figure 1:**
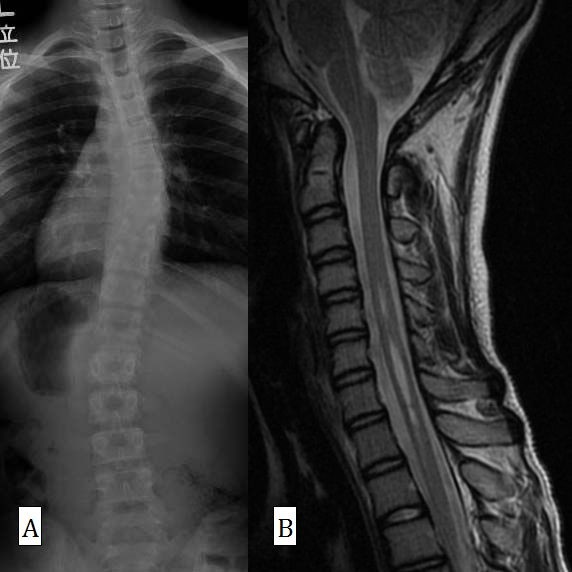
**Posteroanterior radiograph (A) and sagittal MRI (B) in Case 9**. The patient was neurologically normal. The syrinx was spindle type and the space of the cisterna magna was preserved. This patient was not suitable for FMD and was treated using an underarm brace.

Syringomyelia was found in 9 of the 93 patients (9.7%), and 7 of these 9 patients had the Arnold-Chiari malformation. Eight of the 9 patients with syringomyelia had abnormal SAR. Only one patient (Case 9) with syringomyelia, whose subarachnoid space at the foramen magnum was relatively wide, had normal SAR (Fig. 1). The sensitivity of abnormal SAR for syringomyelia was 89%, with 86% specificity, 40% PPV, and 99% NPV (Table 4). Prevalence of abnormal SAR differed significantly between those with scoliosis and syringomyelia (89%) and those with scoliosis without syringomyelia (14%) (P < 0.0001). Focusing the subjects on "idiopathic" scoliosis, the sensitivity of abnormal SAR for syringomyelia was 89%, with 95% specificity, 80% PPV, and 98% NPV (Table 5).

**Table 4:** Abnormal SAR as a predictor of scoliosis with syringomyelia in all patients

	Syringomyelia	No syringomyelia	Total	
Abnormal SAR	8	12	20	PPV 40%
Normal SAR	1	72	73	NPV 99%
Total	9	84	93	
	Sensitivity 89%	Specificity 86%		

NPV, negative predictive value; PPV, positive predictive value; SAR, superficial abdominal reflex

**Table 5:** Abnormal SAR as a predictor of scoliosis with syringomyelia in patients with "idiopathic" scoliosis

	Syringomyelia	No syringomyelia	Total	
Abnormal SAR	8	2	10	PPV 80%
Normal SAR	1	44	45	NPV 98%
Total	9	46	55	
	Sensitivity 89%	Specificity 95%		

"idiopathic" included idiopathic scoliosis and scoliosis with syringomyelia

Of the 9 patients with syringomyelia, 5 with a single major curve and 1 with a double major curve had unilateral hyporeflexia or absence of SAR. Four of the 5 patients with a single major curve showed hyporeflexia or absence of SAR on the same side as the convexity of the curve (Cases 2, 3, 7, 8), but the remaining patient did not (Case 6) (Table 1).

### Curve flexibility of patients with syringomyelia

**Figure 2:**
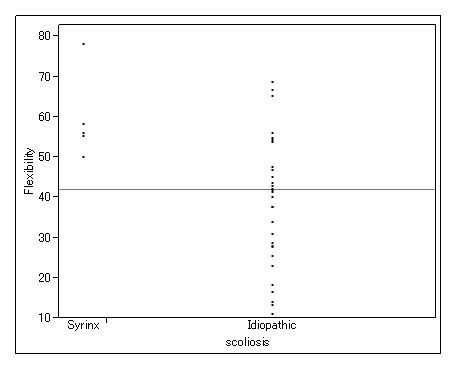
**Distribution of curve flexibility between syringomyelia and idiopathic scoliosis (Cobb angle ≥60°)**.

The mean preoperative Cobb angle for the 6 patients with syringomyelia who had corrective surgery was 78° (range, 56-105°) and mean curve flexibility was 58% (range, 50-78%). Conversely, mean preoperative Cobb angle for patients with idiopathic scoliosis who had corrective surgery was 69° (range, 45-128°) and mean curve flexibility was 44% (range, 11-96%) (Table 6). Curve flexibility was significantly greater with syringomyelia than with idiopathic scoliosis (P = 0.02). Focusing on patients with a Cobb angle ≥ 60°, this difference in curve flexibility between patients with syringomyelia and those with idiopathic scoliosis was much more pronounced (Fig. 2) (P < 0.0001). In the logistic regression model, the area under the ROC curve was 0.79, indicating moderate discriminatory ability of curve flexibility to predict syringomyelia (P = 0.08). The best cut-off value of curve flexibility for syringomyelia was 50% and in this model, the sensitivity was 100%, with 63% specificity, 26% PPV, and 100% NPV (Table 7).

**Table 6:** Curve flexibility of syringomyelia

	Syringomyelia (6 operated cases)	Idiopathic scoliosis (46 operated cases)	P
Gender (% male)	44	4.3	0.02*†
Age (years)	14.5 (8-28)	14.1 (7-21)	0.44
Risser sign	2.8 (0-5)	3.4 (0-5)	0.27
Curve magnitude(°)	78 (56-105)	69 (45-128)	0.17
Curve flexibility (%)	58 (50-78)	44 (11-96)	0.02*

Values are expressed as mean (range).

*Significant difference compared to the idiopathic scoliosis group

†Fisher's exact test

**Table 7:** Flexibility ≥ 50% as a predictor of scoliosis with syringomyelia in "idiopathic" scoliosis underwent operation

	Syringomyelia	No syringomyelia	Total	
Flexibility ≥ 50%	6	17	23	PPV 26%
Flexibility < 50%	0	29	29	NPV 100%
Total	6	46	52	
	Sensitivity 100%	Specificity 63%		

"idiopathic" included idiopathic scoliosis and scoliosis with syringomyelia

### Other indicators for scoliosis with syringomyelia

Four of the 9 patients with syringomyelia were male (44%) and 5 were female (56%). In contrast, 2 of the 46 patients with idiopathic scoliosis were male (4.3%) and 44 were female (96%). There was a significant gender difference between patients with syringomyelia and those with idiopathic scoliosis (Table 6) (P < 0.005). Male sex conferred a relative risk for syringomyelia of 6.5 (CI 2.4-18).

One of the 9 patients with syringomyelia (11%, Case 5) and one patient with idiopathic scoliosis (2.1%) had an atypical left-thoracic curve pattern, but this difference was not significant. (P = 0.3)

### Relationship between syrinx type and neurological abnormality

**Figure 3:**
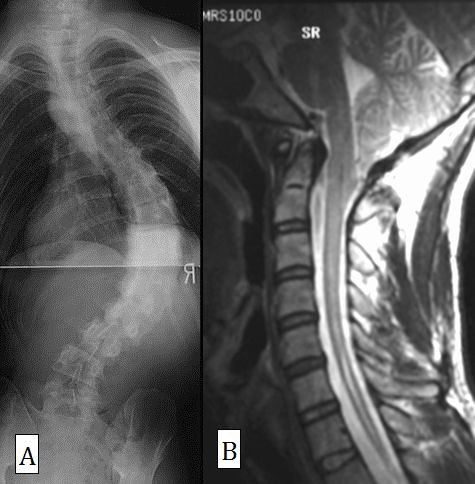
**Posteroanterior radiograph (A) and sagittal MRI (B) in Case 1**. This patient had bilateral hyporeflexia of SAR and declined neurological decompression surgery, but anterior fusion was performed without any neurological complication. The Cobb angle was 90° and curve flexibility was 52%. The syrinx was slit type.

The mean syrinx length was 7.5 vertebrae and the median length was 5 vertebrae. All patients with swelling-type syrinx were neurologically abnormal (Table 8). One patient (Case 1) with bilateral hyporeflexia of SAR declined neurological decompression surgery, but his corrective surgery, for which we selected anterior fusion, was performed without any neurological complication. The syrinx of this patient was slit type (Fig. 3). One patient with normal neurology with the exception of SAR (Case 3) also had slit-type syrinx, which was relatively short (4 vertebrae). Another patient with spindle-type syrinx (Case 9) had completely normal neurological findings and a syrinx length of 4 vertebrae (Fig. 1). However, no significant difference was found between swelling type and the other types (slit or spindle) of syrinx in terms of abnormal SAR or other neurological findings (P = 0.3).

**Table 8:** Relationship between syrinx type and neurological abnormality

Case	1	2	3	4	5	6	7	8	9
**Syrinx type**	**Slit**	**Swelling**	**Slit**	**Swelling**	**Swelling**	**Swelling**	**Swelling**	**Swelling**	**Spindle**
Syrinx level	C3-7	Th6-9	C5-7	C5-8	C5-9	C2-7	C1-L1	C2-Th11	C5-8
Syrinx length (vertebra)	5	4	3	4	5	6	20	17	4
SAR	Bil hypo	R abs	L abs	Bil hypo	L abs	L hypo	L abs	L hypo	Normal
Other neurological findings	Bil Babinski reflex	Hyper of L/E	None	Hyper of L/E	Hypo of L/E	Hyper of ATR	Hypo of L/E	Headache SD of left L/E	None

abs, absence; ATR, Achilles tendon reflex; Bil, bilateral; hyper, hyperreflexia; hypo, hyporeflexia; L, left; L/E, lower extremities; R, right; SAR, superficial abdominal reflex; SD, sensory disturbance

## Discussion

A potential risk of neurologic complications has been reported when corrective surgery is performed for patients with scoliosis and syringomyelia [1-3]. Several studies have found that neurosurgery before corrective surgery reduces the risk of neurological complications and improves or stabilizes the curve, particularly in patients younger than 10 years old [24-29]. Previous reports have agreed with the consensus that MRI should be performed for infantile and juvenile scoliosis [10-12]. However, the necessity of MRI for "adolescent idiopathic" scoliosis remains controversial [11,30-32]. Some studies have suggested that MRI is not indicated in "adolescent idiopathic" scoliosis if the patients are neurologically normal [4,6,7]. Inoue reported that only one of 12 neurologically asymptomatic patients with syringomyelia and scoliosis encountered neurological complications [5]. However, once paraplegia occurred, the prognosis was very poor. We believe corrective surgery for scoliosis should be performed with utmost care so as to minimize the risk of neurological complications. Of the neurological abnormalities thought to indicate syringomyelia, SAR is considered one of the most important [4,9]. However, in early studies, it was unclear how often patients with syringomyelia had normal SAR and how often patients with normal SAR had syringomyelia [13,22]. In Saifuddin's prospective study of 73 patients with "presumed idiopathic scoliosis", the prevalence of abnormal SAR was 11%. Saifuddin concluded that unilateral absence of SAR was a significant sign suggestive of syringomyelia and that bilateral absence of SAR might also be associated with neuraxis anomalies [32]. Morduende reported that 11 patients (15%) of 72 patients with atypical features had abnormality on MRI. In their report, 5 of the 11 patients with abnormal SAR had MRI abnormality and therefore, abnormal SAR had a sensitivity of 45% and PPV of 45%. They concluded that the importance of SAR could not be overemphasized [8,9,31]. However, as a limitation of these studies, they might have differed in their definition of "idiopathic" or "abnormal SAR". If patients without any indicators of syringomyelia were regarded as genuine "idiopathic" cases, this might increase the PPV of SAR. Some studies have reported that absence or hyporeflexia can occur in normal individuals [33]. In the present study, "idiopathic" scoliosis included 46 patients with idiopathic scoliosis and 9 patients with scoliosis and syringomyelia. Data from previous studies regarding asymmetric SAR as abnormal in "idiopathic" scoliosis and those from the present study are listed in Table 9. The sensitivity of abnormal SAR for syringomyelia in the present study was higher than that of previous studies, partly because we included bilateral absence and hyporeflexia of SAR as abnormal signs. When considering SAR as a screening test, sensitivity is more important than PPV, because final diagnosis can be made by MRI. Therefore, it could be recommended that subtle findings such as bilateral absence or hyporeflexia of SAR should be regarded as abnormal to give a useful screening test. In the present study, we found that abnormal SAR (defined as unilateral or bilateral absence of SAR or hyporeflexia as found by a single experienced examiner) yielded 89% sensitivity for syringomyelia. Furthermore, if SAR of a patient with scoliosis is abnormal, the patient has a 90% of probability of being non-idiopathic scoliosis (Table3). If patients with scoliosis had abnormal SAR, we could recommend physicians to check MRI of the whole spine in order to differentiate syringomyelia as well as to check myogenic enzyme in order to differentiate myopathy.

**Table 9:** Diagnostic utility of SAR in "idiopathic" scoliosis

	Sensitivity (%)	Specificity (%)	PPV (%)	NPV (%)	N
Inoue (2004) *	34	97	71	87	250
Saifuddin (2005) *	22	90	25	89	73
Present study	89	95	80	98	55

N, number of patients; NPV, negative predictive value; PPV, positive predictive value; SAR, superficial abdominal reflex * Abnormal SAR means asymmetry

Some studies have reported the relationship between syrinx type and neurological findings or radiographic characteristics [5,34,35]. Inoue reported that all patients with swelling-type syrinx had some abnormal findings and our results supported these findings. In our study, male sex was a risk factor for syringomyelia with a relative risk of 6.5 (CI 2.4-18). However, there was no significant difference in left-thoracic curve pattern between patients with syringomyelia and those with idiopathic scoliosis (P = 0.3). When reporting a good prognostic model for abnormal MRI, Morcuende et al. reported that left-thoracic curve pattern by itself may not be suggestive of MRI abnormality [9]. They concluded that left-thoracic curve pattern was associated with abnormal MRI when combined with neurologic abnormality or a severe curve despite immaturity. Our findings corresponded with theirs.

To our knowledge, there have been no previous reports describing the relationship between curve flexibility and syringomyelia. Some authors have alerted clinicians to the risk of paraplegia due to overcorrection or vulnerability of the spinal cord in corrective surgery for scoliosis with syringomyelia [1,3,23]. Generally, increasing age and larger Cobb angle make the curve less flexible [36]. However, in the current study, curve flexibility of patients with syringomyelia was significantly greater than that of those with idiopathic scoliosis, although mean age and Cobb angle of patients with syringomyelia were larger than those of patients with idiopathic scoliosis (P = 0.02). This suggests that patients with scoliosis and syringomyelia have a more flexible curve than those with idiopathic scoliosis. The logistic regression model was close to being significant (P = 0.08). Based on this logistic regression analysis, curve flexibility ≥50% could be another risk of syringomyelia. Although we cannot explain the pathophysiology of hyperflexibility in syringomyelia, any abnormality in the pyramidal tract would make the curve more flexible in scoliosis with syringomyelia than in idiopathic scoliosis.

## Conclusions

The sensitivity of abnormal SAR for non-idiopathic scoliosis was 38% with 96% specificity, 90% PPV, and 60% NPV. All cases of myopathic scoliosis had bilateral absence of SAR. There were significant differences between patients with syringomyelia and those with idiopathic scoliosis in gender, abnormal neurological findings (SAR), and curve flexibility. The sensitivity of abnormal SAR for syringomyelia in patients with "idiopathic " scoliosis was 89%, with 95% specificity, 80% PPV, and 98% NPV. Curve flexibility ≥50% could be another risk of syringomyelia.

## Competing interests

The authors declare that they have no competing interests.

## Authors' contributions

All authors read and approved the manuscript, which has not been submitted or published anywhere else. TF and MI designed the study. HS, KO, and YN revised it critically. All authors read and approved the final manuscript.
